# Surgical Management of Parapharyngeal Metastases from Thyroid Carcinoma: A Systematic Review and Patient-Level Analysis of the Role of Lateral Neck Dissection

**DOI:** 10.3390/cancers18172799

**Published:** 2026-08-28

**Authors:** Francesco Chiari, Marika Reppucci, Matteo Fermi, Giulia Di Dalmazi, Vincenzo Palatino, Daria Maria Filippini, Giovanni Motta, Giuseppe Mercante, Livio Presutti, Claudio Donadio Caporale, Pierre Guarino

**Affiliations:** 1 Otolaryngology Head and Neck Unit, “Santo Spirito” Hospital, 65124 Pescara, Italy; claudiodonadio.caporale@asl.pe.it (C.D.C.); pierreguarino@hotmail.com (P.G.); 2 Department of Medical and Surgical Sciences (DIMEC), Alma Mater Studiorum-Università di Bologna, 40126 Bologna, Italy; marika.reppucci@gmail.com (M.R.); mercante.giuseppe@gmail.com (G.M.); livio.presutti@unibo.it (L.P.); 3 Department of Otolaryngology-Head and Neck Surgery, IRCCS Azienda Ospedaliero-Universitaria di Bologna, 40126 Bologna, Italy; matteo.fermi.med@gmail.com; 4 Department of Endocrinology, “Santo Spirito” Hospital, 65124 Pescara, Italy; giulia.didalmazi@asl.pe.it; 5 Department of Radiology, “Santo Spirito” Hospital, 65124 Pescara, Italy; vincenzo.palatino@asl.pe.it; 6 Osteoncology, Bone and Soft Tissue Sarcomas and Innovative Therapies Unit, IRCCS Istituto Ortopedico Rizzoli, 40136 Bologna, Italy; d.m.filippini@gmail.com; 7 Head and Neck Surgery Unit, University of Campania “Luigi Vanvitelli”, 81100 Naples, Italy; giovannimotta95@yahoo.com; 8 Department of Innovative Technologies in Medicine & Dentistry, University “G. d’Annunzio” Chieti-Pescara, 66100 Pescara, Italy

**Keywords:** thyroid carcinoma, parapharyngeal space, parapharyngeal metastasis, lateral neck dissection, thyroid surgery

## Abstract

**Background/Objectives**: Parapharyngeal space (PPS) metastases from thyroid carcinoma are rare, and evidence guiding their surgical management, particularly the role of concomitant lateral neck dissection (LND) in patients with a clinically node-negative (cN0) neck, remains limited. This systematic review and patient-level analysis evaluated the clinicopathological characteristics, surgical management, and oncologic outcomes of PPS metastases, with particular emphasis on the management of the cN0 neck. **Methods**: A PRISMA 2020-compliant systematic review of PubMed, Scopus, and the Cochrane Library was performed. Patient-level data were analyzed according to clinical neck status and timing of PPS metastasis presentation. **Results**: Thirty-two studies including 162 patients were analyzed. Papillary thyroid carcinoma accounted for 94.4% of cases, while PPS metastases presented as primary disease in 48.8% and recurrent disease in 51.2% of patients. The transcervical approach (TCA) was the predominant surgical technique (79.1%), whereas LND was performed in 70.2% of patients with available data. Among the 19 patients with a cN0 neck, 10 had a previously undissected lateral neck; of these, 6 underwent elective LND and 4 underwent isolated PPS metastasectomy. No occult cervical lymph node metastases were identified following elective LND. Patients with recurrent PPS metastases had significantly higher overall recurrence (47.5% vs. 13.2%, *p* < 0.001) and disease-specific mortality (22.8% vs. 5.6%, *p* = 0.003) than those with primary PPS metastases. **Conclusions**: TCA remains the reference surgical strategy for PPS metastases from thyroid carcinoma. Although based on a very limited cN0 subgroup, current evidence suggests that routine elective LND may not be necessary in carefully selected patients presenting with isolated PPS metastases and a cN0 neck. Recurrent PPS metastases are associated with poorer oncologic outcomes.

## Simple Summary

Parapharyngeal space metastases from thyroid carcinoma are extremely uncommon, and their optimal surgical management remains poorly defined because the available evidence is limited to small case reports and institutional series. In particular, it remains unclear whether patients without clinically detectable lymph node metastases in the lateral neck should routinely undergo additional neck dissection at the time of surgery. The largest patient-level systematic review currently available was performed to evaluate the clinical characteristics, surgical management, and oncologic outcomes of this rare condition. The findings confirm that the transcervical approach remains the standard surgical technique for most patients and suggest that routine elective lateral neck dissection may not be necessary in carefully selected patients without clinical evidence of lateral neck disease. These results provide the strongest evidence currently available to support individualized surgical decision-making and may help guide future clinical practice and research in this uncommon clinical scenario.

## 1. Introduction

Thyroid carcinoma, particularly papillary thyroid carcinoma (PTC), frequently metastasizes to the cervical lymph nodes, typically involving the central (level VI) and lateral cervical compartments (levels II–V) [1,2,3,4,5,6]. In contrast, metastatic spread beyond these conventional nodal basins is distinctly uncommon.

Parapharyngeal space (PPS) lymph node metastases represent one of the rarest manifestations of regional thyroid cancer spread. Their reported incidence ranges from 0.43% to 2.5% among patients with well-differentiated thyroid carcinoma, and fewer than 200 cases have been reported in the literature over the past three decades, mostly as isolated case reports or small retrospective series [7,8]. The largest surgical series currently includes only 97 patients, whereas the largest oncologic cohort comprises 75 patients, emphasizing the rarity of this clinical entity and the limited evidence available to guide management [5,9].

The occurrence of PPS metastases is explained by the complex lymphatic drainage of the thyroid gland [10]. Owing to their deep anatomical location adjacent to the internal carotid artery, internal jugular vein, and lower cranial nerves, PPS metastases are frequently clinically silent and often detected during postoperative surveillance using contrast-enhanced computed tomography (CT), magnetic resonance imaging (MRI), radioiodine imaging, or positron emission tomography/computed tomography (PET/CT) [3,5,8,11]. Although fine-needle aspiration cytology (FNAC) may establish the diagnosis in selected patients, tissue sampling is frequently challenging because of the anatomical constraints of the PPS [12,13,14]. In selected cases, fine-needle aspiration biopsy (FNAB), particularly when combined with thyroglobulin measurement in the aspirate, represents a valuable adjunct for the differential diagnosis of PPS lesions, allowing reliable distinction between thyroid carcinoma metastases and other retrostyloid neoplasms, including metastatic squamous cell carcinoma, with excellent diagnostic accuracy [15,16].

Complete surgical excision remains the cornerstone of treatment for resectable PPS metastases. However, despite increasing surgical experience and the development of minimally invasive and robotic approaches, several clinically relevant issues remain unresolved [17,18]. In particular, although therapeutic lateral neck dissection (LND) is well established for patients with clinically evident cervical nodal disease, the management of the clinically node-negative (cN0) lateral neck in the presence of PPS metastases remains controversial, especially in previously operated patients, in whom neck dissection may be technically demanding and associated with increased morbidity [4,8,9,19]. This scenario poses a particular clinical challenge because an isolated PPS metastasis does not necessarily imply occult disease within the conventional lateral cervical compartments. Consequently, performing elective LND may expose patients to additional surgical morbidity without a proven oncologic benefit, whereas omitting LND raises concern regarding unrecognized cervical nodal disease and subsequent regional recurrence. This decision is particularly complex in previously operated patients, in whom altered lymphatic drainage and postoperative fibrosis may further complicate both disease presentation and surgical management. Importantly, the 2025 American Thyroid Association (ATA) Guidelines recognize the PPS as a regional nodal compartment distinct from the lateral neck and recommend that these compartments should be evaluated independently [20]. Nevertheless, no evidence-based recommendations are currently available regarding the need for concomitant LND in patients with isolated PPS metastases and a cN0 neck, leaving surgical decision-making largely dependent on individual experience [8,20].

To date, no patient-level systematic review has specifically investigated the role of concomitant LND in patients with PPS metastases from thyroid carcinoma. Therefore, the present systematic review and patient-level analysis aimed to comprehensively evaluate the clinicopathological characteristics, diagnostic work-up, surgical management, adjuvant treatment, and oncologic outcomes of these patients, with particular emphasis on the management of the cN0 neck.

## 2. Materials and Methods

### 2.1. Search Strategy and Information Sources

The present systematic review was conducted according to the PRISMA 2020 statement (Supplementary Materials). A comprehensive literature review was conducted using the PICOTS framework to define the search strategy and inclusion criteria [21,22]. A computerized search was performed across PubMed, Scopus, and Cochrane Library databases to 31 May 2026.

The search strategy combined controlled vocabulary MeSH, when applicable, and free-text terms related to thyroid carcinoma, parapharyngeal metastases, and surgical management. The search syntax was adapted according to the requirements of each database. The following search strategy was used for PubMed: ((“Thyroid Neoplasms”[Mesh] OR “thyroid carcinoma”[Title/Abstract] OR “thyroid cancer”[Title/Abstract] OR “papillary thyroid carcinoma”[Title/Abstract] OR “follicular thyroid carcinoma”[Title/Abstract] OR “medullary thyroid carcinoma”[Title/Abstract] OR “poorly differentiated thyroid carcinoma”[Title/Abstract] OR “anaplastic thyroid carcinoma”[Title/Abstract] OR “differentiated thyroid carcinoma”[Title/Abstract]) AND (“Parapharyngeal Space”[Mesh] OR “parapharyngeal”[Title/Abstract] OR “parapharyngeal space”[Title/Abstract] OR “parapharyngeal metastasis”[Title/Abstract] OR “parapharyngeal lymph node”[Title/Abstract] OR “parapharyngeal node”[Title/Abstract] OR prestyloid[Title/Abstract] OR poststyloid[Title/Abstract])).

The review protocol was prospectively registered in the International Prospective Register of Systematic Reviews (PROSPERO), registration number CRD42026148987.

### 2.2. Study Selection and Data Extraction

After executing the search strategy, the titles and abstracts retrieved were independently screened by F.C. and M.R. Discrepancies regarding citation inclusion were resolved through discussion and consensus. The eligibility of studies was assessed based on the PICOTS criteria, which included:

- Participants: patients with PPS metastases from thyroid carcinoma;
- Intervention: surgical treatment of PPS metastases with or without LND;
- Comparator: comparison according to the clinical neck status at the time of PPS metastasis diagnosis (cN0 vs. cN+) and, among patients with a cN0 neck, according to the performance of concomitant LND;
- Outcomes: clinicopathological characteristics, diagnostic work-up, surgical management, adjuvant treatments, and oncologic outcomes, including recurrence patterns and disease status at the last follow-up (FU);
- Time: until 31 May 2026;
- Study design: retrospective cohort studies, case series, and case reports.

Inclusion criteria were: (1) original studies reporting patients with histologically confirmed PPS metastases from thyroid carcinoma; (2) studies providing individual patient data regarding clinical presentation, diagnostic work-up, surgical management, LND, pathological findings, and/or oncologic outcomes; (3) patients undergoing surgical treatment of the PPS metastasis; (4) retrospective or prospective cohort studies, case series, and case reports; and (5) articles published in English.

Exclusion criteria were: (1) patients with PPS metastases managed non-surgically; (2) studies reporting retropharyngeal lymph node metastases; (3) studies involving primary PPS tumors without metastatic thyroid origin; (4) review articles, editorials, letters, conference abstracts, book chapters, and expert opinions; and (5) studies lacking sufficient clinical or surgical data after full-text review.

**Figure 1:**
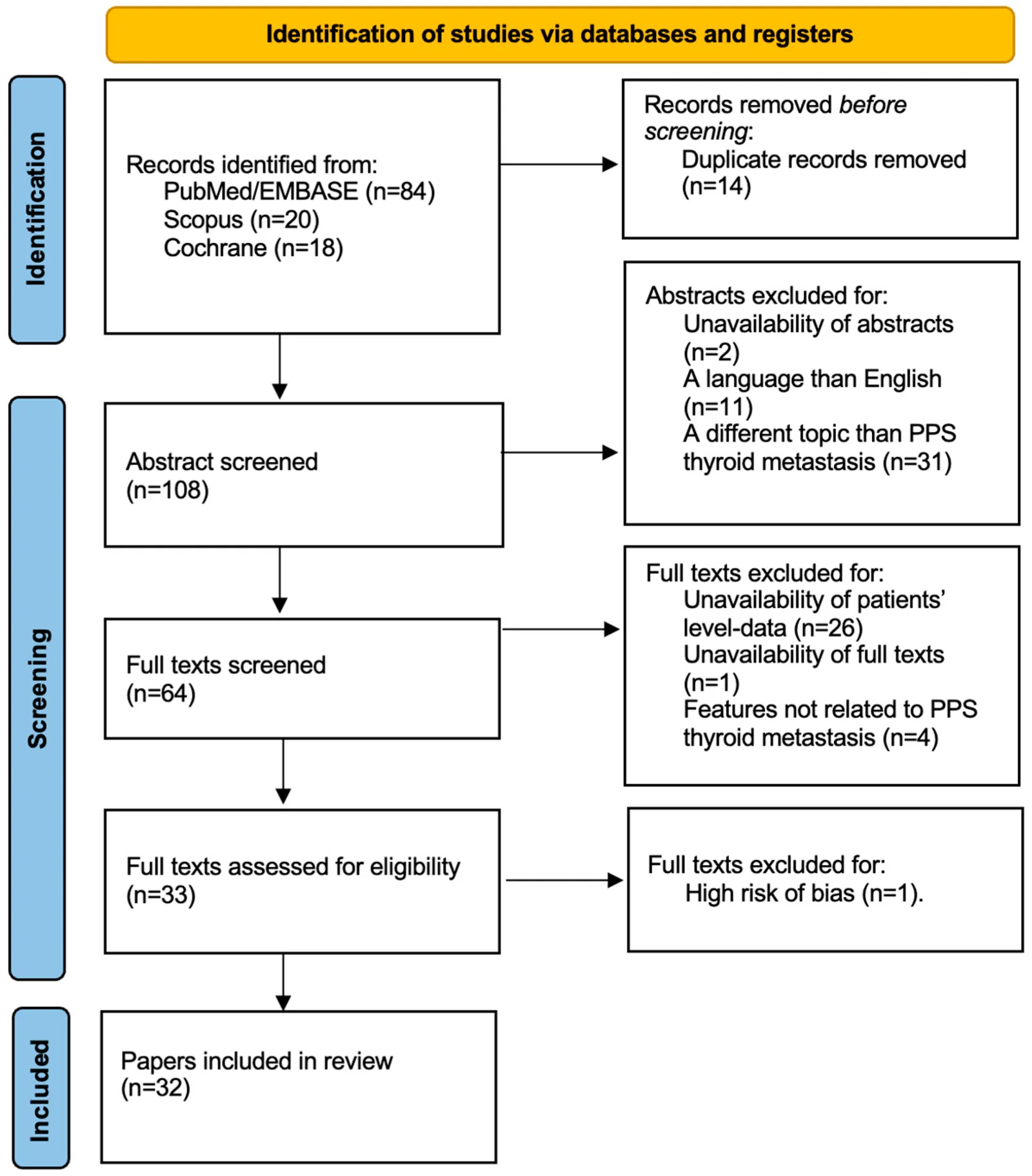
Flow chart of the study.

A manual search of the reference lists of included studies was also performed to identify additional relevant publications. The study selection process is illustrated in the PRISMA flow diagram (Figure 1).

Given the inconsistent anatomical terminology across the literature, lesions were classified according to the anatomical definition provided by the original authors. Therefore, no post hoc reclassification of PPS metastases according to their relationship with the carotid sheath was performed.

### 2.3. Quality Assessment

Methodological quality was independently assessed by two authors (F.C. and M.R.) using the tool proposed by Murad et al. [23] for case series and case reports. Studies were categorized as having low, moderate, or high risk of bias according to the overall score. Articles judged at high risk of bias were excluded from the final analysis (Table 1).

### 2.4. Data Analysis

A descriptive analysis of the extracted data was performed. Continuous variables were reported as mean ± standard deviation (SD), whereas categorical variables were expressed as frequencies and percentages. Given the heterogeneity of reporting across the included studies, percentages were calculated using the number of patients with available data for each variable as the denominator. Consequently, the denominator varied according to data availability.

Comparative analyses were performed according to the clinical neck status at the time of PPS metastasis diagnosis (cN0 versus cN+). Continuous variables were compared using the Mann–Whitney U test, whereas categorical variables were analyzed using Fisher’s exact test. Owing to the limited sample size and the retrospective nature of the included studies, these analyses were considered exploratory.

A predefined subgroup analysis was subsequently performed among patients presenting with a clinically node-negative and previously undissected neck to evaluate the role of elective LND. Given the small number of eligible patients and the heterogeneity of follow-up reporting, this subgroup analysis was limited to descriptive comparisons.

All statistical analyses were conducted using IBM SPSS Statistics version 27.0 (IBM Corp., Armonk, NY, USA). Tumor staging was performed according to the Tumor–Node–Metastasis (TNM) classification system of the 8th edition of the American Joint Committee on Cancer (AJCC) Cancer Staging Manual [46].

## 3. Results

### 3.1. Study Selection

A total of 84, 20, and 18 records were initially identified through the search strategies applied to the PubMed/Embase, Scopus, and Cochrane Library databases, respectively. After the removal of 14 duplicate records, 108 unique articles remained for title and abstract screening. During this phase, 44 records were excluded because of unavailable abstracts (*n* = 2), publication in languages other than English (*n* = 11), or topics unrelated to PPS metastases from thyroid carcinoma (*n* = 31). Consequently, 64 full-text articles were assessed for eligibility. Of these, 31 studies were excluded due to the lack of patient-level data (*n* = 26), unavailable full text (*n* = 1), or because they did not report patients with PPS metastases from thyroid carcinoma (*n* = 4). The remaining 33 studies underwent methodological quality assessment. One study [9] was subsequently excluded because of a high risk of bias (Table 1).

Ultimately, 32 studies comprising 162 patients were included in the final qualitative synthesis and patient-level analysis [1,2,3,5,7,8,11,12,13,14,24,25,26,27,28,29,30,31,32,33,34,35,36,37,38,39,40,41,42,43,44,45]. The study selection process is illustrated in Figure 1, while the characteristics of the included studies are summarized in Table 2.

### 3.2. Patient, Tumor, and PPS Metastasis Characteristics

A total of 162 patients were included in the patient-level analysis. Age was available for 159 patients, with a mean age of 46.2 ± 9.9 years. Sex was reported for 159 patients, including 101 females (63.5%) and 58 males (36.5%).

Histological subtype consisted predominantly of PTC (151/160, 94.4%), followed by MTC (8/160, 5.0%) and FTC (1/159, 0.6%). Primary tumor stage included pT1 in 11 (44.0%), pT2 in 7 (28.0%), pT3a in 2 (8.0%), pT4a in 4 (16.0%), and pT4b in 1 (4.0%). Multifocality status was observed in 5 (27.8%), whereas 13 (72.2%) had unifocal disease. Left-sided tumors accounting for 9 cases (52.9%), right-sided tumors for 6 (35.3%), and isthmic tumors for 2 (11.8%). Previous LND had been performed in 49 of 86 patients (57.0%). Information regarding primary thyroid surgery was available for 151 patients. All patients ultimately underwent total thyroidectomy, either as the initial procedure or as completion thyroidectomy following previous hemithyroidectomy. Information regarding central neck dissection (CND) management at primary treatment was available for 71 patients, of whom 25 (35.2%) underwent central neck dissection. Primary pathological nodal status revealed pN1b disease in 73 (72.3%), pN1a in 17 (16.8%), and pN0 in 11 (10.9%).

At the time of PPS metastasis diagnosis, 79 patients (48.8%) presented with primary disease, whereas 83 (51.2%) developed PPS metastases during FU. Among patients with recurrent PPS metastases, the interval between primary thyroid carcinoma treatment and PPS recurrence presented a mean interval of 95.3 ± 123.1 months. Clinical neck status showed cN1b disease in 63 (76.8%) patients and cN0 disease in 19 (23.2%). A solitary PPS metastasis was identified in 156 patients (96.3%), while multiple lesions in 6 (3.7%) cases. The maximum diameter of the PPS metastasis presented a mean size of 22.5 ± 14.4 mm. Cranial nerve impairment at presentation was reported in 19 patients (24.4%).

Among patients with available information, CT was performed in 51 patients (60.0%), US in 38 (46.3%), PET/TC in 32 (38.6%), and MRI in 32 (37.6%). FNAC was the most frequently adopted diagnostic procedure in 25 cases (83.3%), whereas external biopsy and FNAB were performed in 5 cases 16.7%) and 1 (3.3%) patient, respectively. An additional 53 patients with available data (63.9%) underwent surgical excision without a preoperative biopsy of the PPS lesion.

### 3.3. Surgical Management and Adjuvant Treatments

The transcervical approach (TCA) was the most frequently adopted technique in 68 cases (79.1%), followed by the transparotid approach (TPA) in 6 cases (7.0%), transmandibular approach (TMA) in 5 cases (5.8%), transoral surgery (TOS) in 4 cases (4.7%), combined TCA-TPA approach in 2 cases (2.3%), and combined TCA-TOS in 1 case (1.2%). LND was performed in 59 patients (70.2%), whereas 25 (29.8%) underwent isolated PPS metastasectomy without concomitant LND. Among patients with available data, concomitant CND at the time of PPS metastasectomy was performed in 24 patients (38.7%).

Overall, 110 patients (68.8%) received postoperative radioactive iodine (RAI), whereas adjuvant radiotherapy (RT) was administered in 7 patients (4.4%) and systemic adjuvant treatments were administered in 11 cases (8.4%).

### 3.4. Clinical Neck Status at the Time of PPS Metastasis Diagnosis

Clinical neck status included 19 (23.2%) patients with a cN0 neck and 63 (76.8%) cases with cN+ neck. The comparison of clinicopathological characteristics between the two groups is summarized in Table 3.

Overall, patients with cN0 and cN+ disease showed comparable clinicopathological characteristics. No significant differences were observed regarding sex, histological subtype, timing of PPS metastasis presentation (primary versus recurrent), history of previous LND, or pathological nodal status at primary thyroid surgery. As expected, concomitant LND at the time of PPS surgery was performed significantly more often in patients presenting with cN+ neck than in cN0 (77.8% vs. 42.1%, *p* = 0.005).

Given the limited number of cN0 patients and the retrospective nature of the included studies, these comparisons should be considered exploratory and primarily intended to characterize the study population before the subsequent subgroup analyses.

### 3.5. Management of the Clinically Node-Negative Neck

Among the 19 patients presenting with a cN0 neck at the time of PPS metastasis diagnosis, nine (47.4%) had previously undergone LND, whereas the remaining 10 (52.6%) had a previously undissected neck.

Overall, concomitant LND at the time of PPS metastasectomy was performed in 8 (42.1%) patients, while the remaining 11 patients (57.9%) underwent isolated PPS metastasectomy. However, management differed substantially according to previous cervical treatment. Among patients with a previously undissected neck, 6 (60.0%) underwent concomitant LND, whereas only 2 (22.2%) with a previously dissected neck underwent additional cervical surgery. Conversely, isolated PPS metastasectomy without LND was performed in 4 (40.0%) patients with a previously undissected neck and in 7 (77.8%) patients with a previously dissected neck.

### 3.6. Elective LND in the Previously Undissected cN0 Neck

To better evaluate the role of elective LND, the analysis was restricted to the 10 patients presenting with a cN0 and previously undissected neck. Among these patients, 6 (60.0%) underwent concomitant LND at the time of PPS metastasectomy, whereas 4 (40.0%) underwent isolated PPS metastasectomy without additional cervical surgery. The clinicopathological characteristics and oncologic outcomes of these patients are summarized in Table 4.

No occult cervical lymph node metastases were identified in any of the 6 patients who underwent concomitant LND. During FU, one regional recurrence was documented in one patient undergoing LND, whereas no cervical recurrences were observed in patients managed without LND. Overall recurrence occurred in one patient in each group. All patients with available outcome data were alive without evidence of disease at the last FU.

### 3.7. Oncologic Outcomes According to the Timing of PPS Metastasis Presentation

Oncologic outcomes following PPS metastasectomy according to the timing of PPS metastasis presentation are summarized in Table 5. FU data were available for 68 patients with primary PPS metastases and 78 patients with recurrent disease. Patients presenting with primary PPS metastases had a longer postoperative FU than those with recurrent disease (111.0 ± 60.0 vs. 88.7 ± 55.7 months, *p* = 0.034).

Overall recurrence following PPS metastasectomy was significantly more frequent in patients with recurrent PPS metastases than in those with primary disease (47.5% vs. 13.2%, *p* < 0.001). In contrast, no significant differences were observed in the incidence of local recurrence (11.8% vs. 0.0%, *p* = 0.082), regional recurrence (9.8% vs. 17.2%, *p* = 0.483), or distant recurrence (5.9% vs. 17.2%, *p* = 0.131).

At the last available FU, 65 (90.3%) patients with primary PPS metastases had no evidence of disease, compared with 60 (75.9%) patients with recurrent disease (*p* = 0.031). Conversely, disease-specific mortality was significantly higher among patients with recurrent PPS metastases, occurring in 18 (22.8%) patients compared with 4 (5.6%) patients with primary disease (*p* = 0.003). No significant differences were observed in the proportions of patients alive with persistent disease or those who died from causes unrelated to thyroid carcinoma.

## 4. Discussion

The present systematic review represents, to our knowledge, the largest patient-level analysis specifically investigating the surgical management of PPS metastases from thyroid carcinoma and the first to evaluate the role of concomitant LND according to clinical neck status. By pooling individual data from 32 studies including 162 patients, this study provides the most comprehensive patient-level evidence currently available for this rare clinical entity.

An anatomical and terminological overlap exists between parapharyngeal and lateral retropharyngeal nodal metastases, as lateral retropharyngeal nodes lie close to the medial carotid sheath. The large majority of studies included in the present review reported these lesions simply as PPS or PPS lymph node metastases, without providing sufficient information regarding their precise relationship to the carotid sheath to permit reliable retrospective subclassification. Importantly, the present review was intentionally focused on metastases defined by the original authors as PPS, whereas lesions explicitly reported as retropharyngeal lymph node metastases were excluded. However, a metastatic thyroid carcinoma presenting as a high PPS mass may, in some cases, represent involvement of the upper deep cervical or jugular nodal chain, corresponding to high level II lateral neck disease rather than a truly isolated PPS nodal metastasis. In such circumstances, therapeutic lateral neck dissection is justified. Accordingly, careful assessment of the anatomical relationship of the metastatic node to the carotid sheath and upper jugular chain is essential. Nevertheless, because most primary studies lacked sufficient anatomical detail, we retained the classification provided by the original authors, as post hoc reclassification would require assumptions not supported by the available data.

Surgical management of PPS lesions remains technically demanding because of the deep location of the compartment and its intimate relationship with the internal carotid artery, internal jugular vein, and lower cranial nerves. Consequently, several surgical approaches have been described, including TCA, TPA, TOS, TMA, and combined approaches, each offering specific advantages according to lesion size, anatomical compartment, and neurovascular relationships [10,19].

The predominance of the TCA observed in the present study (79.1%) is consistent with the progressive evolution of the literature and reflects the anatomical characteristics of thyroid carcinoma PPS metastases. Since the earliest reports by Robbins et al. [39], the TCA has remained the preferred surgical corridor because it provides direct exposure of the carotid sheath, lower cranial nerves, and retrostyloid compartment while ensuring adequate vascular control and oncologic resection. Wang et al. [42] reported the successful surgical management of 25 patients, whereas Deng et al. [5] described the largest oncological experience including 75 patients, both confirming that TCA provides satisfactory local control with acceptable morbidity. More recently, Zhang et al. [9] reported the largest dedicated surgical cohort (97 patients), further consolidating the TCA as the reference technique for most PPS metastases. Alternative surgical corridors accounted for only one-fifth of all procedures in the present study. As proposed by Lombardi et al. [34], TPA is primarily indicated for prestyloid or deep parotid lesions, whereas TMA should be reserved for selected tumors with extensive superior extension toward the skull base or inadequate transcervical exposure. Combined approaches, particularly TCA-TPA corridor, have been advocated for large or complex PPS lesions involving both the prestyloid and retrostyloid compartments, providing wider exposure and improved control of the facial nerve and major cervical vessels [45]. Nevertheless, these techniques represented only 3.5% of procedures in this pooled analysis, likely reflecting the clinicopathological characteristics of thyroid carcinoma PPS metastases, which are typically small (mean diameter 22.5 mm), well-circumscribed nodal lesions arising within the retrostyloid compartment and therefore adequately managed through TCA. This observation is not unexpected, as thyroid carcinoma metastases usually present as well-defined lymph node metastases rather than bulky primary PPS tumors, thereby rarely requiring extended surgical exposure. Over the last decade, minimally invasive approaches have progressively expanded the surgical armamentarium for selected PPS lesions. Endoscopic-assisted and transoral techniques have shown encouraging results in carefully selected medially located tumors while reducing surgical morbidity [1,31,47,48]. More recently, transoral robotic surgery has further improved visualization and instrument dexterity within the confined PPS, facilitating access to selected benign and malignant tumors [18,49,50]. However, current evidence remains limited to small institutional series, and no study has demonstrated superior oncologic or functional outcomes compared with the conventional TCA. Therefore, minimally invasive techniques should currently be regarded as complementary options for carefully selected patients rather than replacements for the TCA.

One of the most clinically relevant findings of the present study concerns the management of patients presenting with an isolated PPS metastasis and a cN0 neck. Although this scenario accounted for only 23.2% of patients with available clinical nodal status, cN0 and cN+ patients showed otherwise comparable clinicopathological characteristics, including histological subtype, timing of PPS metastasis, previous LND, and pathological nodal status at primary thyroid surgery, suggesting that differences in surgical management were primarily driven by clinical neck status rather than baseline disease features. These findings provide the first dedicated evidence addressing this clinical scenario and support the concept that the PPS should be considered independently from the lateral neck during surgical decision-making, in line with the 2025 ATA Guidelines [20].

Among the 19 patients presenting with a cN0 neck, 10 had a previously undissected lateral neck, representing the subgroup in whom the indication for elective LND is genuinely relevant. Of these, 6 underwent concomitant LND, whereas 4 were managed with isolated PPS metastasectomy. No occult cervical lymph node metastases were identified following elective LND, while only one regional recurrence occurred during follow-up. Although based on a very limited cohort, these observations question the routine use of elective LND solely because of the presence of an isolated PPS metastasis in the absence of clinical or radiological evidence of lateral neck disease.

These findings may be explained by the unique lymphatic drainage of the thyroid gland. As originally described by Rouvière [24,34], lymphatic vessels arising from the superior thyroid pole may drain directly to the retropharyngeal and parapharyngeal nodal basin through accessory pathways, without necessarily involving the conventional lateral cervical compartments. This anatomical pathway may be particularly relevant for tumors arising from the superior thyroid pole. From a surgical perspective, careful exploration along the medial aspect of the carotid sheath toward the cranial border of level VI, with extension according to intraoperative findings, may therefore be considered in selected patients. Interestingly, 47.4% of cN0 patients had previously undergone LND, lending further support to the hypothesis that postoperative lymphatic remodeling may redirect lymphatic drainage toward the PPS and contribute to the development of isolated PPS recurrence in the absence of persistent disease within the lateral neck [8,11]. Collectively, these observations reinforce the concept that the PPS represents a distinct regional nodal station with its own lymphatic drainage pattern [20].

From a surgical perspective, the decision to perform concomitant LND should always balance the expected oncologic benefit against the morbidity of cervical reoperation. Previous thyroidectomy and neck dissection frequently result in fibrosis and distortion of normal anatomical planes, increasing the technical complexity of surgery and the risk of spinal accessory nerve dysfunction, chyle leak, vascular injury, and wound complications [8,19,51]. In this context, our findings support a selective, risk-adapted strategy in which the extent of cervical surgery is individualized according to preoperative imaging, previous cervical treatment, and the estimated likelihood of occult nodal disease, rather than routinely dictated by the presence of a PPS metastasis [52]. Although these observations require validation in larger multicenter cohorts, they suggest that isolated PPS metastases should not automatically be considered an indication for elective LND in patients with a cN0 lateral neck.

The timing of PPS metastasis also emerged as an important prognostic factor. In the present series, 51.2% of patients developed PPS metastases as recurrent disease after a remarkably long interval from primary thyroid carcinoma treatment (95.3 ± 123.1 months), highlighting the indolent but persistent natural history of thyroid carcinoma. Despite a shorter postoperative follow-up (88.7 ± 55.7 vs. 111.0 ± 60.0 months; *p* = 0.034), patients with recurrent PPS metastases experienced an almost four-fold higher rate of overall recurrence following PPS metastasectomy (47.5% vs. 13.2%; *p* < 0.001) and significantly higher disease-specific mortality (22.8% vs. 5.6%; *p* = 0.003) than those presenting with primary PPS disease. Conversely, 90.3% of patients with primary PPS metastases were disease-free at the last FU compared with 75.9% of those with recurrent disease (*p* = 0.031). Interestingly, these differences occurred despite comparable rates of isolated local, regional, and distant recurrence, suggesting that recurrent PPS metastases may represent a marker of greater cumulative disease burden and more aggressive tumor biology rather than inadequate local control. Similar observations have been reported in the largest contemporary series [5,9,44]. Although complete surgical resection remains the cornerstone of treatment regardless of the timing of presentation, these findings indicate that recurrent PPS metastases identify a subgroup of patients at increased risk of disease progression who may benefit from closer and more intensive surveillance.

The strengths of the present study include the largest patient-level dataset currently available, the comprehensive evaluation of surgical strategies, and the first dedicated analysis addressing the management of the cN0 neck. Nevertheless, several limitations should be acknowledged. All included studies were retrospective, most consisted of case reports or small case series, and heterogeneous reporting resulted in missing data for several variables. Furthermore, the subgroup specifically addressing the role of elective LND was extremely small, including only 10 patients with a cN0 previously undissected neck (6 undergoing LND and 4 managed without LND). This very limited sample size precludes adequately powered statistical comparisons and does not allow definitive conclusions regarding the oncologic safety of omitting elective LND. Therefore, these findings should be considered hypothesis-generating and interpreted with caution until confirmed in larger multicenter cohorts. Moreover, the observed oncologic outcomes cannot be attributed to surgical management alone because of substantial potential confounding. Relevant variables, including tumor histologic subtype, PPS metastasis size, history of previous neck surgery, and postoperative RAI administration and dosage, could not be adequately incorporated into comparative analyses because of incomplete and heterogeneous reporting across the included studies. Therefore, the observed differences in recurrence and mortality should be interpreted as unadjusted associations rather than independent effects. The limited sample size, missing data, and absence of standardized treatment protocols precluded reliable multivariable adjustment for these potential confounders. Despite these limitations, this patient-level analysis provides the highest level of evidence currently available to support surgical decision-making in this uncommon clinical scenario.

## 5. Conclusions

PPS metastases from thyroid carcinoma are rare and may occur either at initial presentation or as recurrent disease, often after previous cervical treatment. Surgical excision remains the mainstay of treatment and provides favorable local control in appropriately selected patients. In patients with a previously undissected cN0 neck, the available evidence does not support routine elective lateral neck dissection. However, given the limited number of such patients and the incomplete anatomical characterization of PPS nodal location in the primary studies, lateral neck management should be individualized according to preoperative evidence of cervical nodal disease and, when assessable, the anatomical relationship of the metastatic node to the carotid sheath and upper jugular nodal chain. Conversely, when the metastatic lesion represents involvement of the upper jugular nodal chain, it should be considered lateral neck disease and managed with therapeutic LND. Further studies with standardized anatomical reporting are required to better define the indications for concomitant lateral neck dissection.

## Figures and Tables

**Table 1:** Selection of articles in order to the risk of bias tool by Murad et al. [23].

Authors	Year	Selection	Ascertainment	Causality	Reporting	Risk of Bias
Aygenc [24]	2002	1	1	1	0	I
Batıoğlu-Karaaltın [25]	2014	1	1	1	0	I
Benet [26]	2015	1	1	1	1	L
Bozza [27]	2009	1	1	1	0	I
sCampagnoli [3]	2023	1	1	1	0	I
Deng [5]	2023	1	1	1	0	I
Desuter [11]	2004	1	1	1	0	I
Erdem [28]	2003	1	1	1	1	L
Ferrario [12]	1995	1	1	1	0	I
Giordano [29]	2015	1	1	1	1	L
Harrathi [30]	2020	1	1	1	1	L
Hyvrard [31]	2024	1	1	1	1	L
Iftikhar [32]	2018	1	1	1	0	I
Jain [33]	2019	1	1	1	0	I
Lajud [1]	2019	1	1	1	1	L
Lombardi [34]	2004	1	1	1	1	L
Masmoudi [35]	2021	1	1	1	1	L
Mishra [36]	2024	1	1	1	1	L
Moritani [7]	2016	1	1	1	1	L
Pearlman [2]	1998	1	1	1	0	I
Pilolli [37]	2016	1	1	1	1	L
Ren [38]	2024	1	1	1	1	L
Robbins [39]	1985	1	1	1	1	L
Sandler [8]	2020	1	1	1	1	L
Saydam [40]	1999	1	1	1	1	L
Sharma [41]	2025	1	1	1	1	L
Thomas [13]	2002	1	1	1	1	L
Tomoda [14]	2005	1	1	1	1	L
Wang [42]	2012	1	1	1	0	I
Wang [43]	2026	1	1	1	1	L
Zhang [9]	2022	1	1	0	0	H
Yu [44]	2019	1	1	1	1	L
Zhao [45]	2024	1	1	1	0	I

*Note*: The methodological quality assessment was based on the tool proposed by Murad et al. [23], which includes eight items grouped into four domains (selection, ascertainment, causality, and reporting). Each domain was considered fulfilled (score = 1) if all items within that domain were satisfied, or not fulfilled (score = 0) otherwise Final results differentiated case reports or series into 3 categories of risk of bias as follows: low risk (L) if all questions of the four domains were matched; intermediate risk (I) if questions of three out of the four domains were matched; high risk (H) if questions of less than three out of the four domains were matched.

**Table 2:** Clinicopathological characteristics, surgical management, and oncologic outcomes of the included studies.

First Author (Year of Publication)	Pts (Males) Mean Age	Histology	T Stage	Previous LND	Primary N Stage	Timing of PPS Diagnosis	Mean nr of PPS Metastasis	Surgical Treatment PPS	LND	Mean Diameter of PPS	**Adjuvant Treatment**	**Recurrence After PPS Removal (Months)**	**Mean FU**
Aygenc (2002) [24]	2 (1) 30y	PTC (2)	NA (2)	Yes (1)	NA	Rec (1) Prim (1)	1	TCA (2)	Yes (2)	40	RAI	NA	NA
Batıoğlu-Karaaltın (2014) [25]	1 (0) 70y	PTC	NA	Yes	NA	Rec	1	TCA	No	100	NA	NA	NA
Benet (2015) [26]	1 (1) 51y	PTC	NA	Yes	NA	Rec	1	TCA- TPA	No	40	RAI–RT-CHT	No	84m
Bozza (2009) [27]	1 (1) 66y	PTC	NA	Yes	pN1b	Rec	1	TCA	No	40	No	No	NA
Campagnoli (2023) [3]	1 (1) 45y	PTC	Pt3a	No	pN1b	Prim	1	TCA	Yes	38	RAI	No	NA
Deng (2023) [5]	75 (32) 40.2y	MTC (5) PTC (70)	NA (75)	NA (75)	pN0 (9) pN1a (12) pN1b (54)	Rec (29) Prim (46)	2 (3) 1 (72)	NA (75)	NA (75)	14.2	No (18) RAI (57)	Yes (24) No (51) NA (75)	151m
Desuter (2004) [11]	3 (NA) NA	PTC (3)	NA (3)	Yes (3)	NA (3)	Rec (3)	1 (3)	TCA (3)	NA	NA (3)	RAI (3)	27 (1)	34.7m
Erdem (2003) [28]	1 (1) 40y	PTC	pT1	No	pN0	Prim	1	TCA	Yes	50	RAI	No	NA
Ferrario (1995) [12]	1 (1) 47y	PTC	pT2	No	pN1a	Prim	1	TCA	Yes	35	RAI	NA	NA
Giordano (2015) [29]	2 (0) 47.5y	PTC	pT1 (1) pT2 (1)	Yes (1) No (1)	pN0 (1) pN1a (1)	Prim (1) Rec (1)	1 (2)	TCA (2)	Yes (1) No (1)	NA (2)	RAI (2) RT (1)	No	17.5m
Harrathi (2020) [30]	1 (1) 50y	PTC	pT1	No	pN2b	Prim	1	TOS	Yes	20	RAI	No	NA
Hyvrard (2024) [31]	1 (0) 42y	MTC	NA	Yes	NA	Rec	1	TCA–TOS	Yes	40	No	No	1m
Jain (2019) [33]	1 (1) 38y	PTC	NA	Yes	NA	Rec	1	TCA–TPA	Yes	35	RAI	No	14m
Iftikhar (2018) [32]	1 (1) 38	PTC	NA	No	NA	Prim	1	TCA	Yes	NA	RAI	No	49m
Lajud (2019) [1]	1 (1) 66y	PTC	NA	No	NA	Rec	1	TCA-TPA	No	40	No	No	16m
Lombardi (2004) [34]	2 (1) 46y	PTC (1) NA (1)	pT2 (1) pT4b (1)	No (2)	pN1a (2)	Prim (2)	1 (2)	TCA (2)	Yes (1) No (1)	60	RAI (2) CHT (1)	No (2)	NA
Masmoudi (2021) [35]	1 (0) 62y	PTC	pT1	Yes	pN1b	Rec	1	TCA	Yes	30	RAI	No	3m
Mishra (2024) [36]	1 (0) 30y	PTC	pT2	No	pN1b	Prim	1	TMA	Yes	27	RAI	No	NA
Moritani (2016) [7]	22 (5) 63.4y	PTC (22)	pT1 (2) NA (20)	No (2) Yes (22)	pN1b (2) NA (20)	Prim (2) Rec (20)	2 (1) 1 (21)	TCA (22)	Yes (22)	28	RAI (8) No (14)	Yes (11) No (11) NA (22)	67m
Pearlman (1988) [2]	1 (1) 69y	PTC	pT1	No	pN1b	Prim	1	TMA	Yes	NA	No	NA	NA
Pilolli (2016) [37]	2 (0) 47.5y	PTC (2)	NA	Yes (2)	NA	Rec (2)	1 (2)	TCA (2)	Yes (1) No (1)	33.5	No	No	22m
Ren (2024) [38]	1 (0) 52y	PTC	pT4a	Yes	NA	Rec	1	TCA	Yes	NA	No	No	NA
Robbins (1985) [39]	1 (0) 62y	PTC	NA	No	NA	Prim	1	TCA	No	60	NA	No	60m
Sandler (2020) [8]	1 (0) 55y	PTC	NA	Yes	NA	Rec	1	TMA	No	86	RT	No	21m
Saydam (1999) [40]	1 (0) 54y	NA	pT1	No	pN1b	Prim	1	TCA	Yes	NA	No	No	36m
Sharma (2025) [41]	1 (0) 24y	PTC	pT1	No	pN1b	Prim	1	TCA	No	53	RAI	108	108m
Thomas (2002) [13]	1 (1) 46y	PTC	pT1	No	pN1b	Prim	1	TCA	Yes	60	RAI	No	12m
Tomoda (2005) [14]	1 (0) 58y	PTC	pT2	No	pN1b	Prim	1	TCA	Yes	60	RAI	No	NA
Wang (2012) [42]	25 (5) 45.3y	PTC (22) MTC (2) FTC (1)	NA (25)	Yes (12) No (13)	NA (25)	Rec (16) Prim (9)	1 (25)	TCA (24) TMA (1)	No (13) Yes (12)	NA (25)	RAI (16) RT (2) CHT (1) No (9)	Yes (11) No (14)	33.7m
Wang (2026) [43]	1 (0) 72y	PTC	pT4a	No	pN1b	Prim	1	TOS	Yes	30	RAI	NA	NA
Yu (2019) [44]	6 (3) 50.7y	PTC (6)	pT1(2) pT2 (1) pT3a (1) pT4a (1) NA (1)	Yes (1) No (5)	pN1b (5) NA (1)	Rec (1) Prim (5)	1 (5) 2 (1)	TCA (6)	Yes (5) No (1)	24	RAI (6)	No (6)	46m
Zhao (2024) [45]	1 (0) 58y	PTC	pT4a	Yes	pN1a	Rec	2	NA	NO	1	RAI CHT	NA	NA

*Abbreviations*: CHT = chemotherapy; FNAB = fine needle aspiration biopsy; FNAC = fine needle aspiration cytology; FTC = follicular thyroid cancer; LND = lateral neck dissection; MTC = medullary thyroid cancer; NA = not available; Prim = primary; Pts = patients; PTC = papillary thyroid cancer; RAI = radioactive iodine; Rec = recurrence; RT = radiotherapy; TC = transcervical; TMA = transmandibular; TOS = transoral surgery; TPA = transparotid; y = year.

**Table 3:** Comparison of clinicopathological characteristics according to clinical neck status at the time of parapharyngeal space metastasis diagnosis.

Variable	cN0	cN+	*p* Value
Female sex, n/N (%)	10/19 (52.6%)	48/63 (76.2%)	0.082
PTC histology, n/N (%)	17/18 (94.4%)	59/62 (95.2%)	1.000
Recurrent PPS metastasis, n/N (%)	10/19 (52.6%)	39/63 (61.9%)	0.595
Previous neck dissection, n/N (%)	9/19 (47.4%)	35/63 (55.6%)	0.605
Primary pN-positive disease, n/N (%)	8/9 (88.9%)	16/17 (94.1%)	1.000
LND at PPS surgery, n/N (%)	8/19 (42.1%)	49/63 (77.8%)	0.005

*Abbreviations*: cN = clinical node status; LND = lateral neck dissection; PPS = parapharyngeal space; PTC = papillary thyroid cancer. *Note*: Percentages were calculated using the number of patients with available data for each variable.

**Table 4:** Oncologic outcomes according to elective LND in patients with a cN0 previously undissected neck.

Variable	LND (n = 6)	No LND (n = 4)
Occult cervical metastases, n/N (%)	0/6 (0.0%)	--
Any recurrence, n/N (%)	1/5 (20.0%)	1/4 (25.0%)
Regional neck recurrence, n/N (%)	1/5 (20.0%)	0/4 (0.0%)
Patients alive without evidence of disease at last FU, n/N (%)	5/5 (100%)	4/4 (100%)

*Abbreviations*: FU = follow-up; LND = lateral neck dissection. *Note*: Percentages were calculated using the number of patients with available FU data.

**Table 5:** Oncologic outcomes following PPS metastasectomy according to the timing of presentation.

Variable	Primary PPS Metastasis (*n* = 79)	Recurrent PPS Metastasis (*n* = 83)	*p* Value
FU after PPS metastasectomy, months (mean ± SD)	111.0 ± 60.0	88.7 ± 55.7	0.034
Any recurrence, n/N (%)	10/76 (13.2%)	38/80 (47.5%)	<0.001
Local recurrence, n/N (%)	0/29 (0.0%)	6/51 (11.8%)	0.082
Regional recurrence, n/N (%)	5/29 (17.2%)	5/51 (9.8%)	0.483
Distant recurrence, n/N (%)	5/29 (17.2%)	3/51 (5.9%)	0.131
NED, n/N (%)	65/72 (90.3%)	60/79 (75.9%)	0.031
AWD, n/N (%)	3/72 (4.2%)	0/79 (0.0%)	0.106
DOD, n/N (%)	4/72 (5.6%)	18/79 (22.8%)	0.003
DOC, n/N (%)	0/72 (0.0%)	1/79 (1.3%)	1.000

*Abbreviations*: AWD = alive with disease; DOC = died of other causes; DOD = died of disease; NED = no evidence of disease; PPS = parapharyngeal space; SD, standard deviation. *Note*: Percentages were calculated using the number of patients with available data for each variable. Continuous variables were compared using the Mann–Whitney U test, whereas categorical variables were compared using sFisher’s exact test.

## Data Availability

All data generated or analyzed during this study are included in this published article.

## Supplementary Materials

The following supporting information can be downloaded at: https://www.mdpi.com/article/10.3390/cancers18172799/s1.

## Abbreviations

The following abbreviations are used in this manuscript:

AJCC	American Joint Committee on Cancer
ATA	American Thyroid Association
AWD	Alive with Disease
cENE	Clinical Extranodal Extension
cN	Clinical Nodal Status
CHT	Chemotherapy
CND	Central Neck Dissection
CT	Computed Tomography
DOC	Died of Other Causes
DOD	Died of Disease
FNAB	Fine-Needle Aspiration Biopsy
FNAC	Fine-Needle Aspiration Cytology
FU	Follow-up
FTC	Follicular Thyroid Carcinoma
LND	Lateral Neck Dissection
MRI	Magnetic Resonance Imaging
NED	No Evidence of Disease
PET/CT	Positron Emission Tomography/Computed Tomograph
PPS	Parapharyngeal Space
PROSPERO	International Prospective Register of Systematic Reviews
PRISMA	Preferred Reporting Items for Systematic Reviews and Meta-Analyses
PTC	Papillary Thyroid Carcinoma
RAI	Radioactive Iodine
RT	Radiotherapy
SD	Standard Deviation
TCA	Transcervical Approach
TMA	Transmandibular Approach
TNM	Tumor–Node–Metastasis
TOS	Transoral Surgery
TPA	Transparotid Approach
